# Teething during sleep: Ultrastructural analysis of pharyngeal muscle and cuticular grinder during the molt in *Caenorhabditis elegans*

**DOI:** 10.1371/journal.pone.0233059

**Published:** 2020-05-20

**Authors:** Alessandro P. Sparacio, Nicholas F. Trojanowski, Karen Snetselaar, Matthew D. Nelson, David M. Raizen

**Affiliations:** 1 Department of Biology, Saint Joseph’s University, Philadelphia, Pennsylvania, United States of America; 2 Department of Neurology, University of Pennsylvania, Philadelphia, Pennsylvania, United States of America; 3 Department of Biology, Brandeis University, Waltham, Massachusetts, United States of America; East Carolina University, UNITED STATES

## Abstract

Complex extracellular structures exist throughout phylogeny, but the dynamics of their formation and dissolution are often opaque. One example is the pharyngeal grinder of the nematode *Caenorhabditis elegans*, an extracellular structure that ruptures bacteria during feeding. During each larval transition stage, called lethargus, the grinder is replaced with one of a larger size. Here, we characterize at the ultrastructural level the deconstruction of the larval grinder and the construction of the adult grinder during the fourth larval stage (L4)-to-adult transition. Early in L4 lethargus, pharyngeal muscle cells trans-differentiate from contractile to secretory cells, as evidenced by the appearance of clear and dense core vesicles and disruptions in sarcomere organization. This is followed, within minutes, by the dissolution of the L4 grinder and the formation and maturation of the adult grinder. Components of the nascent adult grinder are deposited basally, and are separated from the dissolving larval grinder by a visible apical layer. The complete grinder is a lamellated extracellular matrix comprised of five layers. Following grinder formation, pharyngeal muscle cells regain ultrastructural contractile properties, and muscle contractions resume. Our findings add to our understanding of how complex extracellular structures assemble and dissemble.

## Introduction

Complex extracellular structures are found in all lineages of organisms and are responsible for much of the vast diversity in structure seen throughout biology. Such extracellular biological wonders include biofilms of bacteria, exoskeletons of arthropods and molluscs, and the plumage, hair, teeth, and nails of vertebrates. In animals, these structures are composed of acellular matrices of solid structure deposited on the apical side of polarized epithelial cells [1]. Apical extracellular matrices (aECMs) consist of lipids, proteins, and polysaccharides, and they vary in composition and morphology [2, 3]. aECMs serve as physical frameworks for cells, are capable of stimulating vital biochemical and mechanical signals required for tissue morphogenesis, differentiation, and homeostasis, and are the first line of mechanical barriers between the organism and its environment [4–6].

A particular type of exoskeleton, the cuticle, turns over when a new and often larger structure replaces the old one during molting, [7–9]. Molting can be categorized as saltational or continuous. Saltational molts are observed in taxa with tanned cuticles, which are stiff exoskeletons composed of cross-linked chitin and sclerotin. Animals employing saltational molting expand body size only during molts, and not between molts. In contrast, continuous molting occurs in animals with flexible cuticles, allowing growth between molts, such as in the case of the collagenous body coverings of nematodes. Nematodes exhibit both types of molting: saltational molting occurs in the cuticle that lines the pharynx whereas the cuticle lining the body allows for growth between molts, making this molting process continuous [10].

While the nematode body cuticle consists largely of collagens [11], the chemical composition of the nematode pharyngeal cuticle remains poorly-defined. The pharynx is a muscular feeding organ which begins at the buccal cavity. It is comprised of a procorpus, anterior bulb, isthmus and terminal bulb and it terminates posteriorly at the pharyngeal-intestinal valve [12] (**S1 Fig**). Pharyngeal contractions cause posterior movement of alimentary contents, including bacterial food, towards the intestine [13, 14]. Mechanical digestion occurs in the pharyngeal lumen, whose cuticle likely contains the hardy biomaterial chitin [15] as well as possibly functional amyloid [16]. Contraction of the pharyngeal muscles rotates the grinder, an ornate extracellular organ in the terminal bulb consisting of three radially arranged, interconnecting plates of teeth affixed to the apical surfaces of posterior pharyngeal muscle cells 6 and 7 (pm6, pm7). The movement of these teeth causes ingested bacteria to rupture, and allows the passage of macromolecules for digestion and absorption in the intestine.

Like the body wall cuticle, the pharyngeal lining (including the grinder) is remodeled during each molt. Observations made with the light microscope reveal a salutatory growth pattern for the grinder [16]. This developmental process occurs during lethargus, a sleep-like behaviorally quiescent period that occurs at transitions between larval stages and between the fourth larval stage (L4) and adulthood [17]. Successful reconstruction of the grinder is critical for fitness, since mutants with abnormal grinders display stunted growth due to inadequate feeding and nutrition [13, 15, 16, 18, 19]. However, little is understood about the processes responsible for removing the old grinder and creating the new one during each molt.

To better understand the molting process and the mechanisms of extracellular organ formation in general, we visualized at the ultrastructural level the grinder and pharyngeal tissue during the L4-to-adult lethargus period. Through precise staging, we found that the adult cuticle is a multi-lamellated structure assembled in a sequential manner, and that its assembly is temporally coupled to the degradation of the prior-stage cuticle. Reconstruction of the pharyngeal cuticle is a result of pharyngeal muscle cell trans-differentiation, whereby the contractile cellular organization is disrupted by epithelial/secretory features. Moreover, we identify the metalloendopeptidase NAS-6 as a required component for larval grinder dissolution but not for adult grinder formation.

## Materials and methods

### *C*. *elegans* maintenance and strains

Experiments were performed on hermaphrodites. The wild-type strain was variety Bristol, strain N2, and the strain VH1312 had the genotype *nas-6*(*hd108*). Both strains were cultivated at 20ºC. N2 animals were fed the OP50 derivative DA837 [20], while VH1312 animals were fed HB101 *E*. *coli* [21], which supports better growth in feeding defective mutants [22]. Worms were cultivated and fed bacteria on the agar surface of 5.5 cm diameter plastic Petri dishes. The agar was made with nematode growth medium (NGM).

### Temporal staging for microscopy

Animals were staged using a stereomicroscope by observing pharyngeal pumping, a behavior that ceases at the beginning of lethargus [17, 23] and resumes at the end, as well as based on the morphology of the developing vulva [24]. Before lethargus, pharyngeal pumping was visualized at 40X total magnification, and the sub-stage of L4 was determined by the animal’s vulval morphology. We used differential interference contrast microscopy (DIC) at 1000X total magnification to identify L4 animals that were between the L4.5 and L4.7 sub-stages of vulval development. During this time, the paired vulF cells are in close proximity to one another but have not yet made contacts [24]. These developmental events occur prior to the initiation of lethargus [25]. These pre-lethargus animals were selected and fixed for electron microscopy.

We used the following procedure to stage animals during lethargus with five-minute precision: Pumping late-L4 stage animals were transferred in groups of five to an agar surface previously seeded with bacteria and placed in a 20°C incubator. Every five minutes, the animals’ pharyngeal pumping status was inspected at 80X on a stereomicroscope. Animals that had stopped pumping since the prior observation period were transferred individually to a fresh NGM agar plate. Pumping cessation (PC) is an indicator of an animal’s entrance into the L4-adult lethargus period, which was designated as t = 0 minutes. These non-pumping animals were then aged in a 20°C incubator for the following durations prior to fixation and processing for EM: t = 5, 10, 15, 30, 45, 60, and 150 minutes.

To identify first-day and eight-day old adults, actively pumping L4 animals were picked onto freshly seeded plates and fixed the following day (day-1 adult) or nine days later (day-8 adult), the latter of which was transferred several times to avoid crowding.

Due to feeding difficulties, *nas-6* mutant animals reached adulthood with low penetrance and many adults died within 1–2 days, as previously noted [19]. We therefore selected the healthiest *nas-6* mutant adults based on size and active pharyngeal pumping and their stage was confirmed by vulval morphology at 1000X prior to fixation.

### Light microscopy

L4.7 candidate animals were picked from an uncrowded stock plate and transferred to a freshly seeded plate. Immobilization pads of 10% agarose dissolved in M9 were prepared on glass microscope slides, followed by the addition of 0.5 μL of 0.1 μm diameter polystyrene microbeads, as described [26]. Microbeads cause physical confinement, as opposed to chemical anesthetics which paralyze the animals. We used microbead-based immobilization in order to minimize changes to cellular morphology during the preparation [26]. A single L4 animal was transferred to the drop of microbeads onto which a cover slip was gently positioned. The animal was visualized at 1000x total magnification with a DIC oil immersion objective with numerical aperture 1.30 and 10x ocular lenses on an Olympus BX51 light microscope. When the L4.7 stage was confirmed by DIC, the cover slip was gently removed, and the animal was transferred using a fixative-containing glass Pasteur pipette into a well of primary fixative within a Pyrex spot plate.

### Time-lapse videography

A wild type L4 animal whose pharyngeal pumping had slowed but had not yet stopped was chosen for video analysis. Time-lapse videography of grinder development from start to end of the L4-to-adult molt was performed with a 100x oil immersion objective lens using microbead immobilization [26]. Images were acquired using Olympus cellSens© image capture software. The software supported a maximum movie file duration of five minutes per capture. Therefore, we captured 28 successive, 5-minute movie files over a 140 minute period with no lapsed time between individual movie files. A time lapse movie was generated using Windows Movie Maker, and a timestamp was appended using Adobe After Effects CC 2017.

### Transmission Electron Microscopy (TEM)

#### Fixation

Animals were prepared for ultrastructural visualization with slight modifications of the Immersion Fixation for Structure protocol described by Hall et al., 2012 [27]. Animals were immersed in a primary fixative solution containing 0.8% glutaraldehyde and 0.8% osmium tetroxide in 0.1 M sodium cacodylate buffer (pH 7.4) at room temperature (22-25ºC). Animals were cross-sectioned at their posterior end with a 45º microsurgical knife tool (Electron Microscopy Sciences, catalog # 72047–45) and placed on ice for 1 hour. Subsequently, specimens were washed in 0.1 M sodium cacodylate buffer (pH 7.4) at 4ºC, followed by secondary fixation in 2% osmium tetroxide in 0.1 M sodium cacodylate buffer (pH 7.4) at 4ºC for 12–16 hours. Following fixation, the specimens were washed 3–4 times in 0.1 M sodium cacodylate buffer (pH 7.4) at 4ºC and then embedded in 2% agarose blocks that were cured for 20–30 minutes at 4ºC. Thereafter, dehydration was performed according to the following schedule at room temperature: 50% ethanol for 10 minutes; 70% ethanol for 10 minutes; 90% ethanol for 10 minutes; three incubations of 100% ethanol for 10 minutes; 50:50 ethanol:acetone for 15 minutes; and, two incubations of 100% acetone for 15 minutes. The fixation and dehydration steps were performed in a multi-well Pyrex spot plate, with the dehydration steps occurring under the cover of a large plastic Petri dish lid to mitigate evaporation. For tissue infiltration and embedment, an Ultrabed Low Viscosity Epoxy Embedding Kit was used (Electron Microscopy Sciences, catalog # 14310). Tissue infiltration was performed in scintillation vials on a rotator at room temperature according to the following schedule: 3:1 acetone:resin for 45 minutes; 1:3 acetone:resin for 45 minutes; three incubations in 100% resin, first for 4 hours, second for 4 hours, and last for 8–12 hours. Each step occurred under the cover of a large plastic Petri dish lid. Differing from Hall et al. 2012, where propylene oxide was used as the organic solvent during infiltration, we used acetone to satisfactory effect. Lastly, samples were embedded again in freshly prepared 100% resin and left to cure for 48 hours at 60ºC in an aluminum dish mold.

#### Sectioning

Embedded specimens were selected for sectioning based on the positioning of the head/neck region and body angle of a worm in the resin molt. Specimens with a straightened head/neck region and planar position parallel to the resin cast bottom were mounted for processing, whereas samples that were bent, kinked, or set at an off angle (i.e., 5-10º perpendicular to the resin surface) were not. Specimens were cut in a square shape from the cast with a jeweler’s saw. These square, specimen blocks were superglued onto a mount and subsequently trimmed for sectioning.

Sectioning was performed on a Leica Ultracut UCT Ultramicrotome. Specimens were thick-sectioned at 3 μm with a glass knife, placed on slides and stained with 0.5% toluidine blue in sodium metaborate for 75 seconds. Slides were viewed on an Olympus BX51 light microscope (LM) at 1000x oil immersion to determine the depth of sectioning. We defined optimal depth by visualization of two of the three following anatomical features: (i) the terminal bulb lumen, (ii) pharyngeal-intestinal valve (VPI) cells, and/or (iii) the proximal intestinal lumen (**S1 Fig**).

Ultrathin sections were cut using a diamond knife and placed on a 0.25% Formvar supporting film prepared on a domino rack and left to dry overnight.

Grids were post-stained for 5 minutes at room temperature in a saturated solution of uranyl acetate, followed by 5 minutes in Reynold’s (1963) lead citrate. Images were captured on a JEOL-1010 transmission electron microscope, processed using Adobe Photoshop CC 2017 (contrast adjustments and montage preparation) and pseudocolored using Adobe Illustrator CC 2019.

#### Micrograph analysis

We made measurements of cuticular features using Fiji image processing package [28]. Tooth length was measured as the distance between the apical tip of each tooth and the median point between the adjoining basal cuticle curves. Tooth width was measured by drawing a line across the widest part of the tooth. Overall grinder diameter was measured by drawing a line across the portion of the terminal bulb that contained grinder cuticle (**S2 Fig**).

For pseudocoloring, differentiation of grinder lamellae was based on clear structural margins and appearance, which included assessment of stain intensities (i.e. electron density) as well as evaluation of characteristic patterning.

## Results and discussion

We defined structural features and described the events underlying the replacement of the L4 grinder with the adult grinder, during the fourth lethargus stage. To accomplish this with temporal precision, we devised a staging scheme based on the time at which the animal stopped pharyngeal pumping (see Methods for detail).

### The *C*. *elegans* pharyngeal grinder is reconstructed during L4-to-adult lethargus

**Fig 1:**
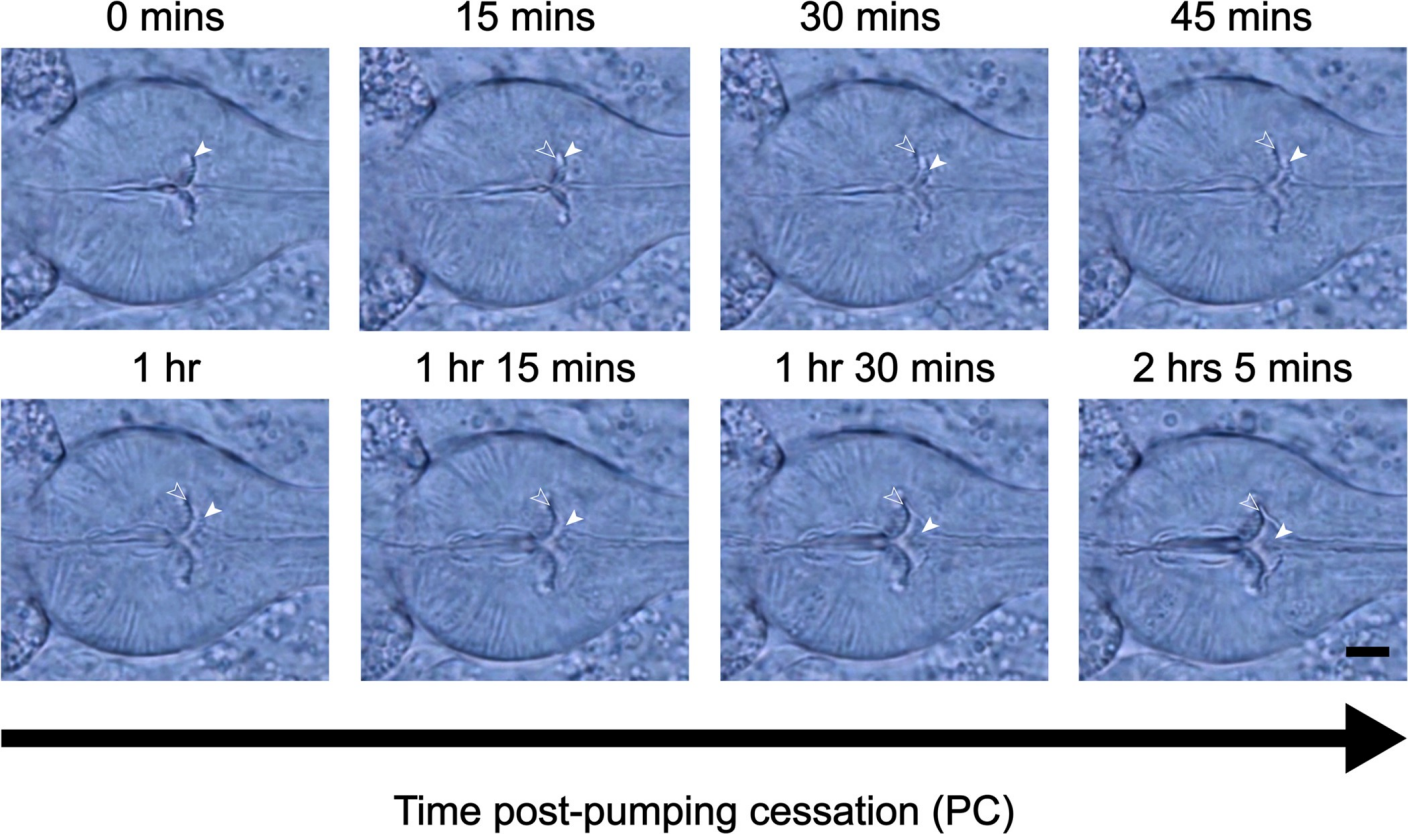
*C*. *elegans* pharyngeal grinder is morphologically changed during the L4-to-adult molt. **The** DIC Images at different times after pumping cessation. Anterior is to the right. The terminal bulb containing the grinder is centered in the field of view. Filled white arrows point to the L4 grinder and unfilled arrows point to the nascent adult grinder. Scale bar = 5 μm.

Some aspects of grinder development are resolvable at the light microscope level. Therefore, we used time-lapse video DIC microscopy to identify key time points in grinder development to stage for electron microscopy (**Fig 1** and **S1 Video**) (see Methods for detail). We used pumping cessation (PC), which occurs at the beginning of lethargus, to establish a time zero (t = 0) for grinder development during L4 lethargus. As previously reported [16], time-lapse videography centered on the terminal bulb showed displacement and dissolution of the L4 grinder anteriorly, concurrent with the formation of the adult grinder posteriorly (**Fig 1** and **S1 Video**). A new grinder was completed within 120-minutes post-PC at 20ºC. In the time window between 15- and 30-minutes post-PC, we observed two distinct grinders, smaller anterior and larger posterior, while 45 to 120-minutes post-PC, the larval grinder became increasingly amorphous as it moved into the luminal space. During this time, the adult grinder took on an increasingly defined shape. Between 1-hour post-PC and the resumption of pharyngeal contractions, we saw no further changes to the new grinder at the level of the light microscope (**Fig 1** and **S1 Video**).

### The *C*. *elegans* pharyngeal grinder comprises five extracellular layers, which are larger in the adult stage than in the L4 stage

**Fig 2:**
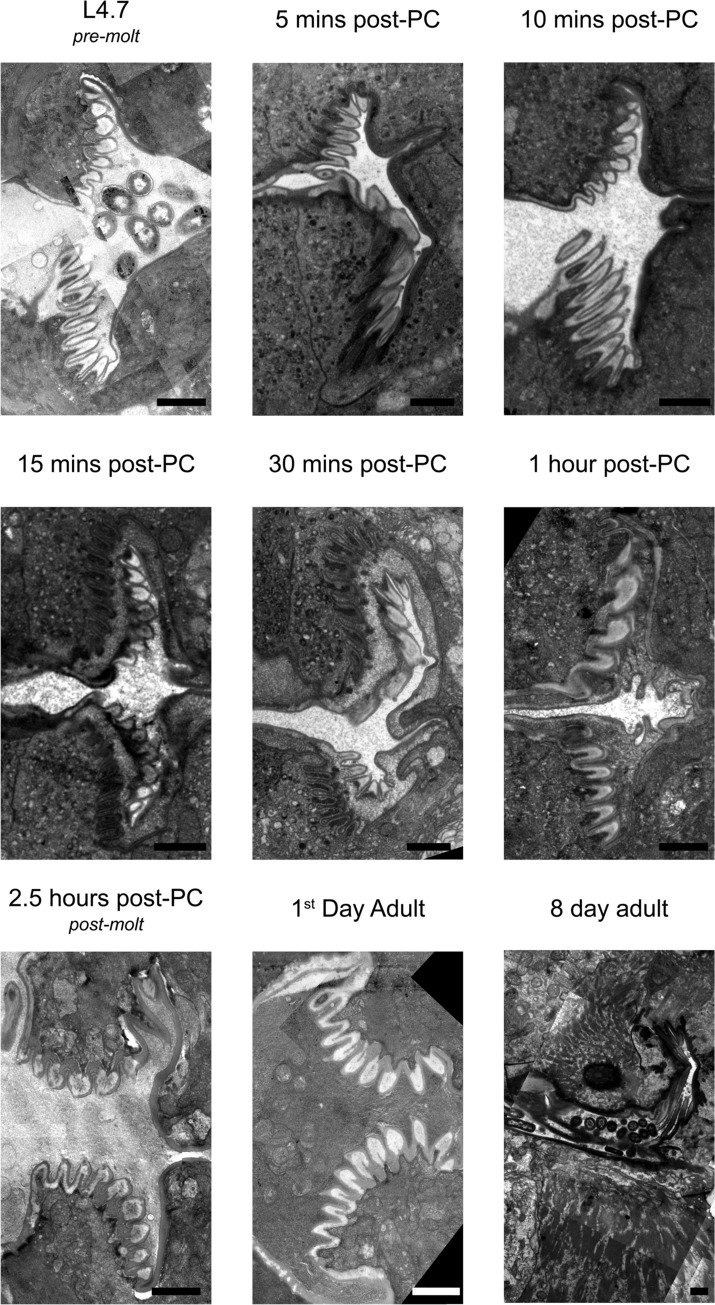
Transmission electron micrographs of the medial terminal bulb region in animals at various indicated times post pumping cessation (PC) or at the pre-lethargus L4.7 stage. The larval pharyngeal cuticle is separated from pharyngeal muscle cells and degraded through the first hour of the molt. At the same time, pharyngeal muscle cells adopt an epithelial secretory character, distinct from their contractile cell character, to synthesize and assemble an adult grinder. Anterior to the right. Scale bars = 1 μm.

**Fig 3:**
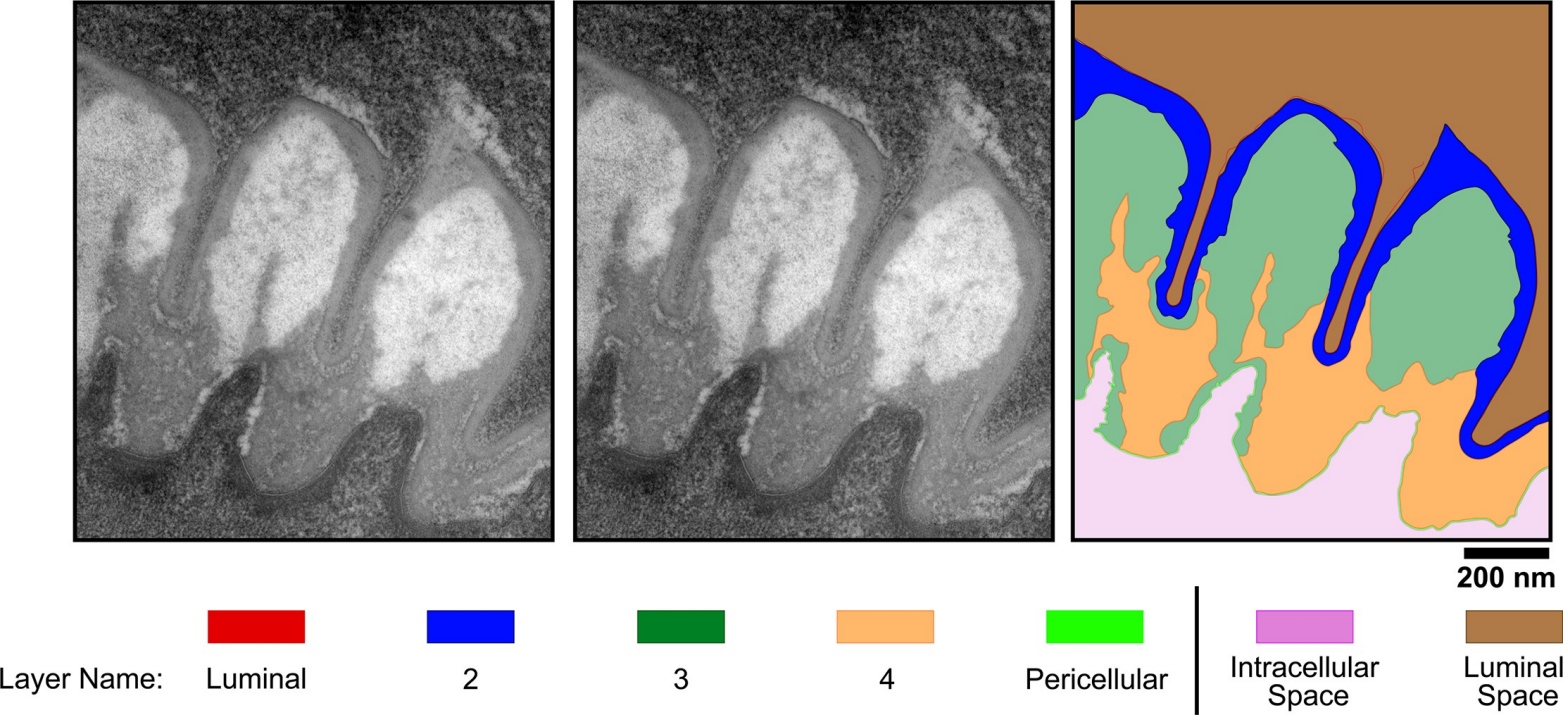
The grinder is composed of five layers of extracellular components. (A) Transmission electron micrograph of the grinder in an active 1^st^ day adult animal. (B) Pseudocoloring of image shown in (A). (C) Cartoon line drawing generated from psuedocoloring shown in (B). The names of the five layers and of the intracellular and luminal spaces are indicated in the color key. Scale bar = 200 nm.

To visualize the ultrastructural changes that occur in the pharynx during lethargus, we then turned to TEM. We first examined the mature grinder of a wild type day-1 adult animal via electron microscopy (**Fig 2 –bottom, middle panel**). The mature, adult grinder consists of tooth-like structures that protrude into the pharyngeal lumen. For each tooth, we can discern five layers of extracellular matrix, which are distinguished by position, degree of electron density, and width. We name these layers in order, starting with the layer closest to the lumen (layer 1) and ending with the one most lateral to the lumen (layer 5) (**Fig 3**). We refer to layer 1 as the luminal layer because it is in direct contact with the luminal contents and to layer 5 as the pericellular layer because it contacts pharyngeal muscle cell membranes. The luminal layer (layer 1) is thin (6.2 ± 1.6 nm) and more electron dense than layer 2. The luminal layer contacts bacterial lysates, and therefore is likely resistant to enzymes that hydrolyze protein and/or carbohydrate. Layer 3, which forms the bulk of the middle of each tooth, has the lowest electron density in comparison to the other layers. Layer 4, making up the base of each tooth, has an electron density similar to layer 2 but it is less uniform in appearance and has a marble-like quality. The pericellular layer (layer 5) has a low electron density cable-like appearance and is slightly wider (9.9 ± 2.3 nm) than the luminal layer. We rank the degree of electron density, from least to most as follows: layer 3 < layer 2 < layer 4 < layer 5 < layer 1.

Next, we visualized the grinder of an active L4 animal before lethargus (**Fig 2 –Top, left panel**). Qualitatively, the five layers are indistinguishable between the active L4 and adult life stages. However, quantitatively, there are differences in the width of each layer, individual tooth width and height, and in the diameter of the entire grinder. As previously observed using light microscopy [16], and consistent with our current light microscopic observations, the extracellular matrix layers increase in size from the L4 to the adult stage (**Fig 1**). The entire grinder width is increased by ~20% between the L4 and adult stages. Two possibilities, which are not mutually exclusive, can explain this difference in size. The first is that the total number of teeth increase between the L4 and adult stage. The second possibility is that the overall growth of the structure is the consequence of growth of the grinder’s individual teeth. We used our electron micrographs to measure individual components of the grinder at L4 and adulthood (**S2 Fig**). Layers 1–5 enlarged by 36%, 26%, 17%, and 19%, and 36% respectively (**Table 1**). The height and width of individual teeth increased by 11% and 26%, respectively (**Table 2**). While we could not count every tooth in a given grinder, our observations suggest that the overall larger size of the grinder in the adult stage relative to the L4 stage is explained by growth of individual teeth, rather than by an increase in their number. It follows that there is likely a one-to-one correspondence of L4 teeth to adult teeth, such that the adult grinder is a larger replica of the L4 grinder.

**Table 1:** Measurements of individual grinder layers at the active L4.7 and 1st day adult stages.

Layer Name	Average Width (nm)	StDev (nm)	Average Width (nm)	StDev (nm)
	L4.7		1-day Adult	
**Luminal**	4.00	1.33	6.24	1.6
**2**	32.6	13.3	44	31.2
**3**	199.3	67.7	240.9	40.1
**4**	85.9	45.3	105.9	82.4
**Pericellular**	6.31	1.01	9.87	2.3

**Table 2:** Measurements of the grinder at the active L4.7 and adult stages.

Layer Name	Measurement (nm)	StDev (nm)	Measurement (nm)	StDev (nm)
	L4.7		1-day Adult	
**Tooth Width**	278	72	374	130
**Tooth Height**	720	227	809	145
**Grinder Diameter**	6111	-	7644	-

In *Pristionchus pacificus* and other members of the nematode family Diplogastridae, there is no terminal bulb grinder. *P*. *pacificus* does however have teeth at the anterior end of the pharynx, which differ depending on whether the animal is in its bacteriovorous feeding mode or predatory feeding mode. Like the *C*. *elegans* grinder, *P*. *pacificus* anterior teeth are continuous with the cuticle lining the pharyngeal cuticle and therefore may have a similar ultrastructure to that of the *C*. *elegans* grinder [29, 30].

### Pharyngeal muscle cells adopt a secretory phenotype during lethargus

**Fig 4:**
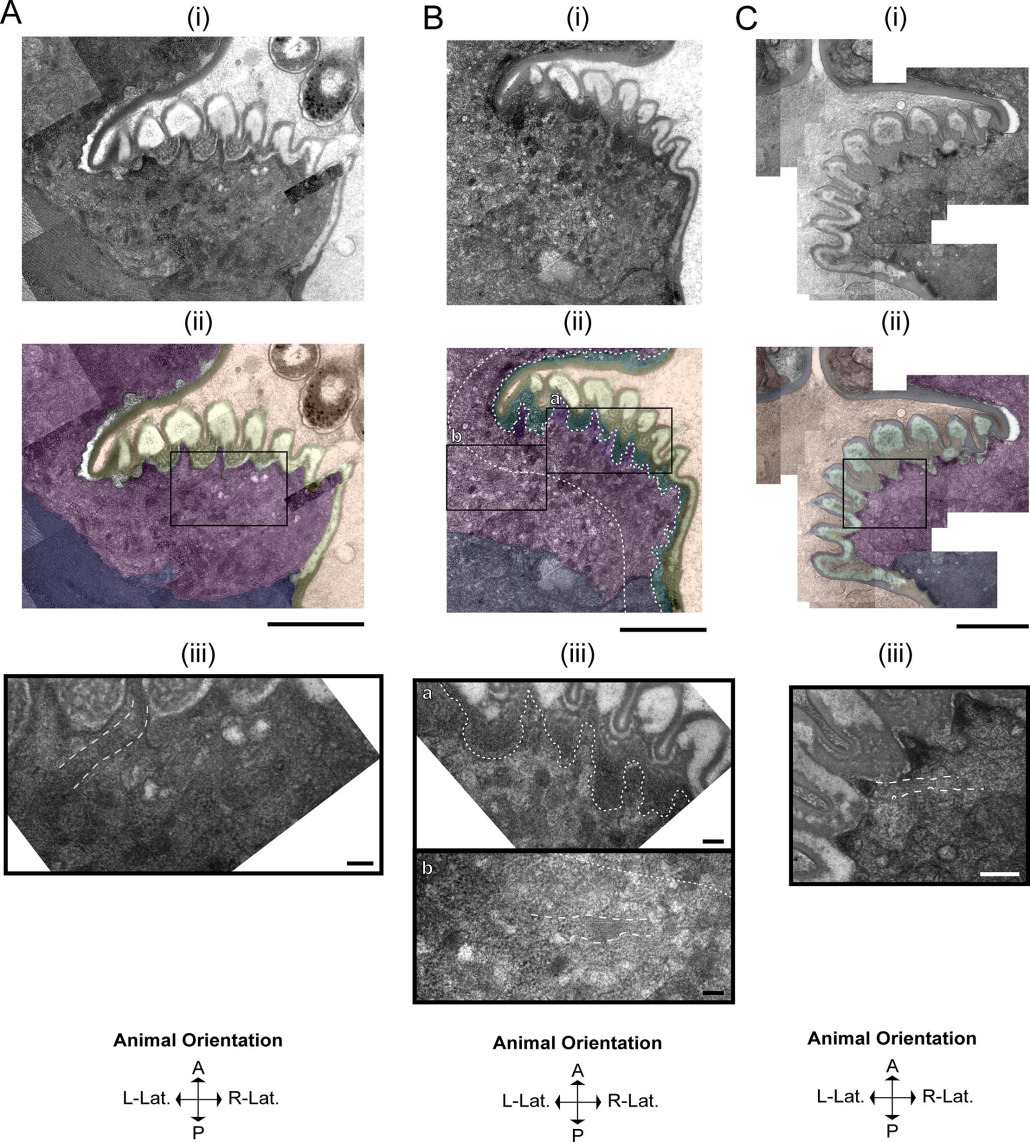
Pharyngeal myoepithelial cells pm6 and pm7 transdifferentiate during lethargus into secretory cells. TEM Image of the grinder at the active L4.7 stage (A), 10 min post-PC stage (B), and 2.5 hr post-PC stage (C). For each stage, the top row (i) shows the TEM, the middle row (ii) shows pseudocoloring of the TEM, and the bottom row (iii) shows an area of pm6 subjacent to the grinder. In the pseudocolored images (ii), light brown denotes the terminal bulb lumen, yellow denotes the L4 grinder, cyan denotes the separation space between the larval grinder and pharyngeal myoepithelial cells, purple denotes pm6 papillae, pink denotes pm6, and navy denotes pm7. Scale bar = 1 μm in the top 2 images in all three panels A-C. (Aiii) L4.7 animals have uninterrupted muscle striations (highlighted by white dash boundaries) connected to the plasma membrane below the apical membranes of pm6 and pm7. Scale bar = 100 nm in Aiii. (Bii and Biii) In the 10-min post-PC terminal bulb, the area between the apical membranes of of pm6 and pm7 and ~500 nm below the apical membranes is the interruption zone or IZ (bounded by white dashed lines. High magnification view of the area outlined in Bii shows interruption of muscle striations; dense core vesicles predominate in this area of pm6. Scale bar = 100 nm for Biiib (b) High magnification view of the box shown in b ii, showing muscle striations (in between parallel white dashed lines) in an area located posterior of the IZ in pm6. Scale bars = 100 nm. (Ciii) High magnification view of the box in (Cii), showing restoration of cell-to-cuticle and muscle striation attachments to the basal-side of the adult grinder (muscle striations are bounded by white dashed lines). Scale bar = 200 nm in Ciii.

As described above, the replacement of the larval grinder with the adult grinder occurs rapidly, raising the question: what cellular processes promote simultaneous grinder disassembly and construction? Using TEM, we find that these dynamics are underpinned by modulation of cell fate, whereby pharyngeal myoepithelial cells of the terminal bulb temporarily transdifferentiate from a contractile to a secretory form (**Fig 4**). In active L4 and adult stages, muscle striations are visible subjacent to the grinder in pm6 cells and the cuticle in pm7 cells (**Fig 4A and 4C**). At the onset of lethargus, we observe a surge of vesicular activity in pm6 and pm7, which is evident at 5 minutes post-PC but drastically reduced by 60 minutes. During this hour, intracellular vesicles congregate in the areas directly beneath the L4 grinder in pm6 and on the medial sides of pm6 and pm7 parallel to the alimentary lumen (**Fig 4B**). In contrast to the active stages, during lethargus these vesicles interrupt the muscle striations (**Fig 4B**). We term the intracellular regions of disassembled muscle morphology the “interruption zone” (IZ). The IZ includes pm6 cellular projections into the individual teeth, the intracellular areas directly subjacent to these projections, and the intracellular region bordering the lumen lining in pm7 cells. The IZ has disorganized muscle structure throughout lethargus including the 60-minute PC time point, but shows normal organization at the 2.5 hours post-PC time point (**Fig 4C**).

Trojanowski *et al* reported that direct optogenetic stimulation of the pharyngeal muscle, which elicits robust contractions in the active L4 and in the adult stages, does not result in contraction or in intracellular calcium elevation during lethargus [31]. Our ultrastructural observations suggest that the quiescence of pharyngeal contractions during lethargus is partially due to interruption of sarcomere structure. We propose that devolution of muscle structure, i.e., loss of intracellular structural continuity, is conducive to vesicular transport required for rapid pharyngeal cuticle turnover.

### Vesicle morphology changes during the progression of lethargus

**Fig 5:**
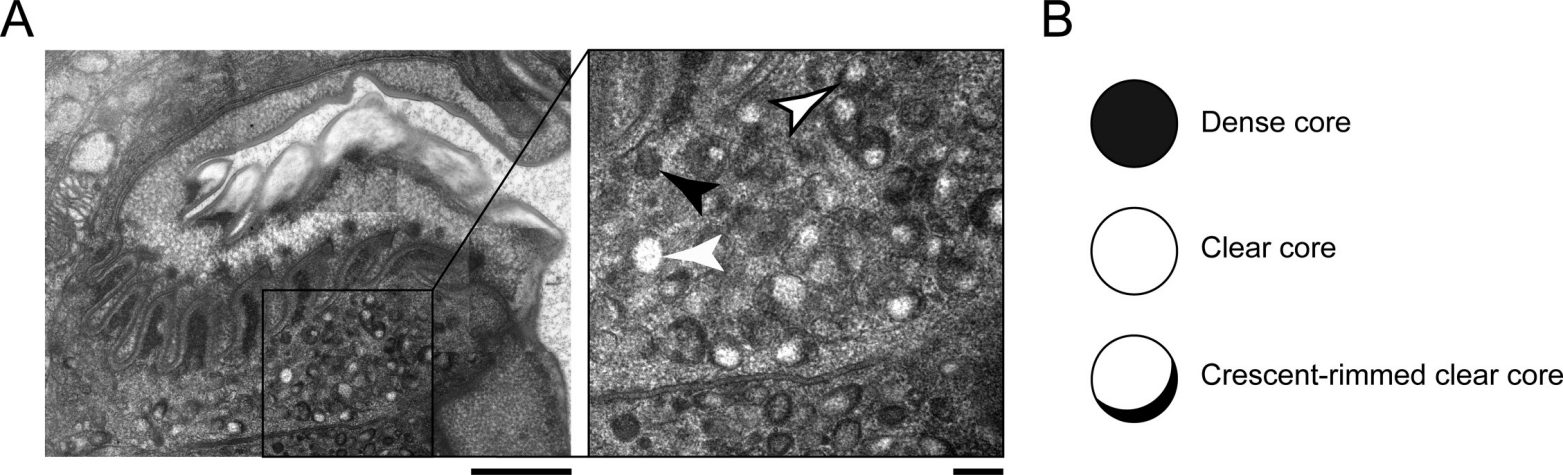
Vesicular morphology changes during lethargus, coincident with L4 grinder dissolution and adult grinder construction. (A) Transmission electron micrograph of an animal 30 minutes post-pumping cessation (PC). Insert: High magnification view of vesicular constituents subjacent to the grinder in pm6, as well as part of pm7. The top-half of the image shows the shed L4 grinder as well as the developing adult grinder, and the bottom-half shows pm6 and pm7 cells. The pm6 and pm7 cells are separated by plasma membranes. A dense core vesicle, denoted by a black arrowhead, appears set to fuse with the plasma membrane. A clear core vesicle is denoted by a white arrowhead, and a crescent-rimmed clear core vesicle is denoted by a black-outlined white arrowhead. Scale bars = 1 μm for the left image and 200 nm for the right image. (B) Cartoon representations of the vesicular types observed in terminal bulb muscle cells during lethargus.

The predominant vesicle type 10-minutes post-PC had an electron-dense core. These dense core vesicles (DCVs) congregated in pm6 and pm7 cells immediately subjacent to the grinder (**Fig 5)**. The pm6 cells appear to physically support the grinder teeth while the pm7 cells physically support the pharyngeal cuticle lining the pharynx. Hence, it is likely that the pm6 cells secrete the grinder (with all its teeth) whereas pm7 and other pharyngeal myoepithelial cells secrete the cuticle lining other parts of the pharyngeal lumen. Because at 10-minutes post-PC the L4 grinder detaches from pm6 and pm7, we suggest that these DCVs carry machinery for enzymatic and/or chemical digestion of the L4 grinder attachment to pm6 and the pharyngeal cuticle to pm7. At time points between 15 and 60 minutes post-PC, we observed primarily clear core vesicles, some of which had a rim of electron-dense material. These clear vesicles appear at the same time that the adult grinder matures. By young adulthood, vesicular activity is diminished, with only rare clear vesicles. Based on the timing of their appearance, we propose that the clear vesicles, with and without a dense rim, contain building blocks for the nascent adult cuticle.

### The L4 grinder is progressively digested basally and the remnants are swallowed

**Fig 6:**
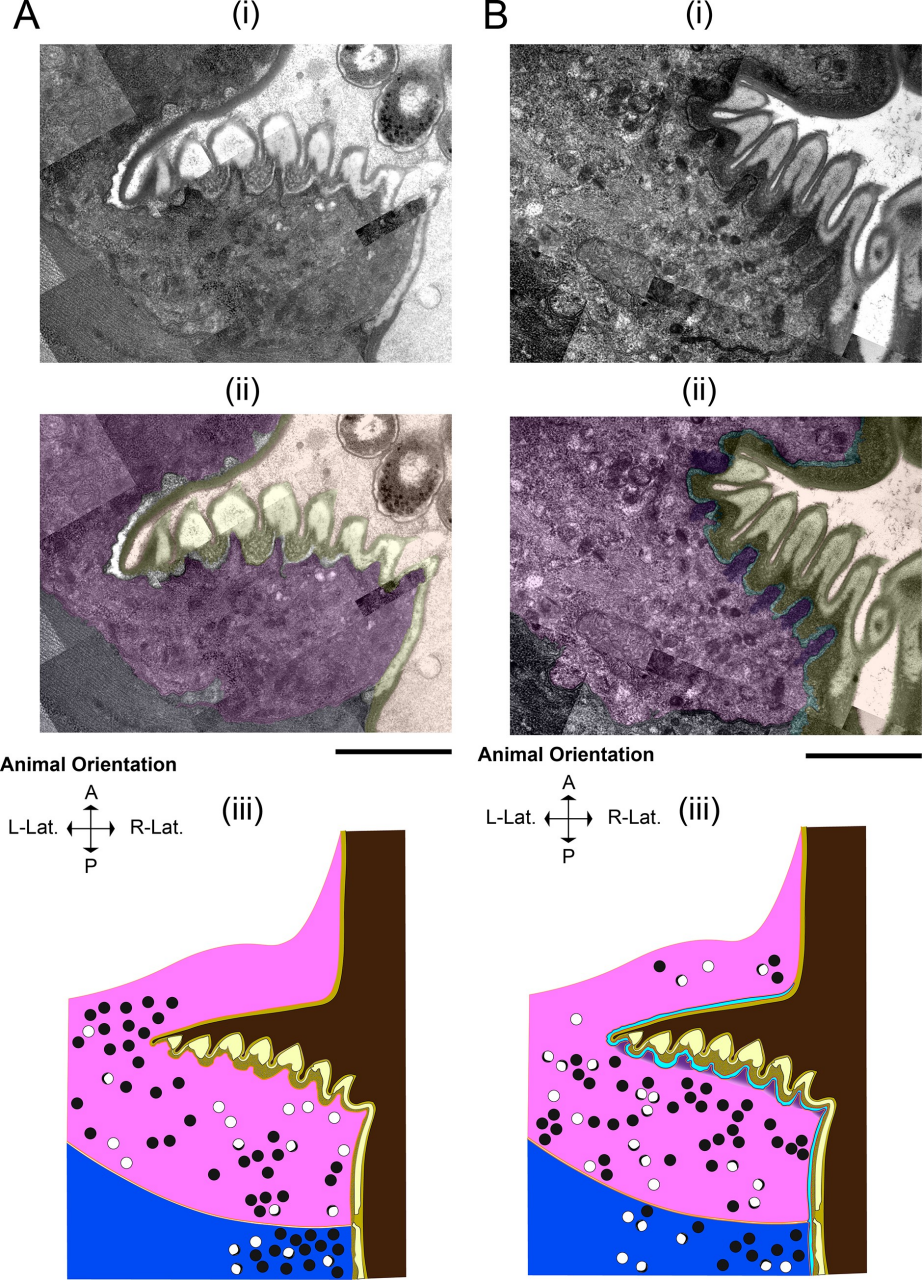
L4 grinder degradation begins rapidly after feeding cessation, coincident with the appearance of abundant dense core vesicles. (A) Active L4.7 and (B) five min post-PC. (i) TEM of the terminal bulb lumen, L4 pharyngeal grinder, pharynx muscle cell 6 (pm6,) and part of pharynx muscle cell 7 (pm7). (ii) Pseudocoloring, light brown denotes the terminal bulb lumen, yellow denotes the L4 pharyngeal grinder, pink denotes pm6, purple denotes pm6 papillae, red denotes the bottom boundary of L4 pharyngeal grinder, and navy denotes pm7. (iii) Cartoon using the color scheme used in (ii) with the addition of orange denoting plasma membranes. In the L4.7 terminal bulb, vesicular activity is evident in both pm6 and pm7, though there are more vesicles in the 5 min post-PC pm6 and pm7 cells. Anterior is up. Scale bar = 1 μm.

**Fig 7:**
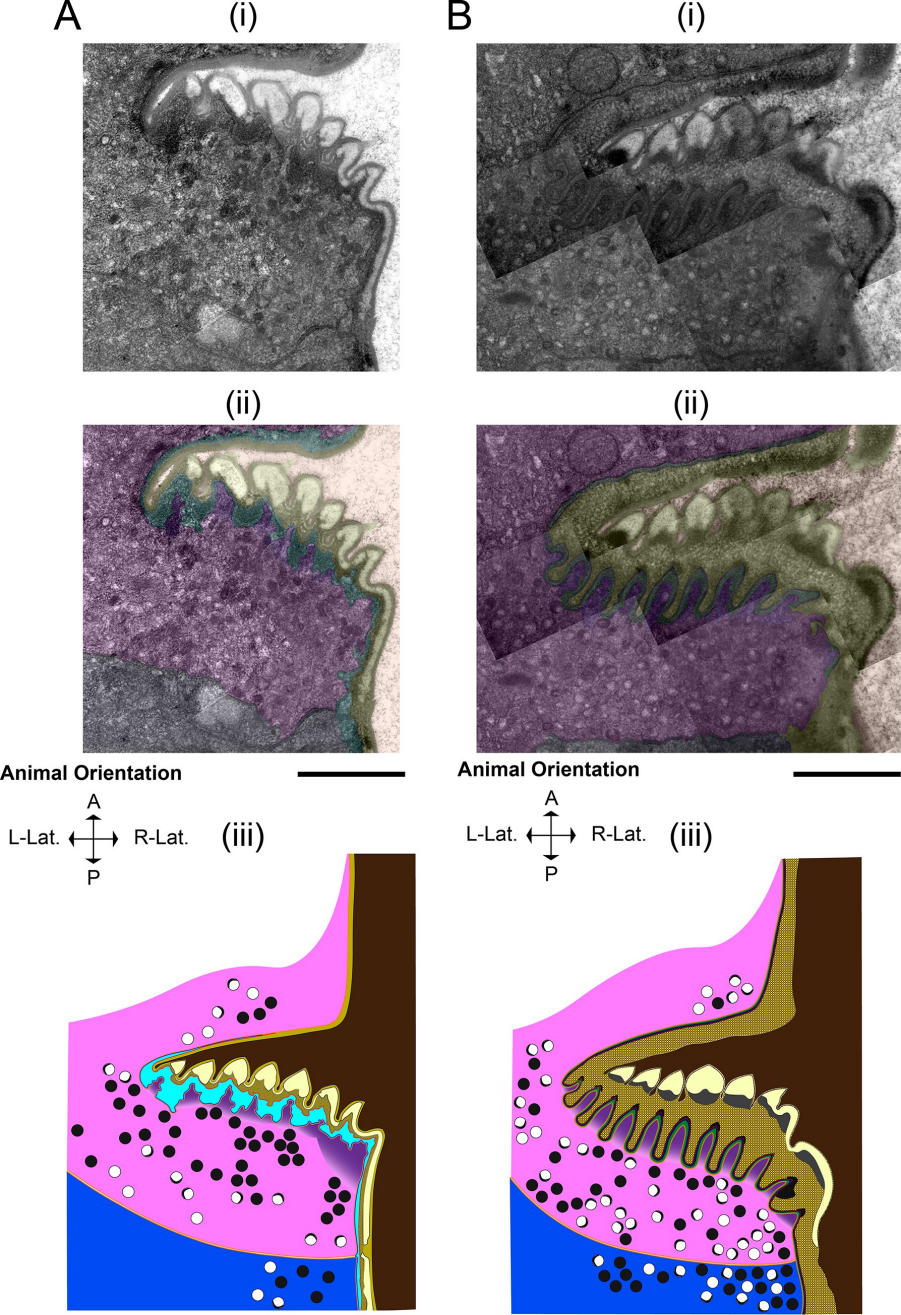
The adult grinder is formed through the sequential addition of layers, coincident to displacement and degradation of the L4 grinder. (A) 10 minutes post-PC and (B) 15 min post-PC. (i) TEM of the terminal bulb. (ii) Pseudocoloring. Light brown denotes the terminal bulb lumen, yellow denotes the L4 pharyngeal grinder, red denotes the bottom boundary of L4 pharyngeal grinder, cyan denotes the separation space, blue denotes the upper boundary of pm6, purple denotes pm6 papillae, pink denotes pm6, and navy denoting pm7. (iii) Cartoon using the color scheme used in (ii) with the addition of plasma membranes shown in orange. Anterior is up. Scale bar = 1 μm.

Maturation of the adult grinder occurs at the same time as digestion and clearance of the L4 grinder. In an active L4.7 animal (**Fig 6A**) (approximately 3 hours before feeding cessation,) pm6 cells have projections into the base of the grinder teeth, and the remainder of the cells’ surfaces contour to the L4 cuticle’s basal surface. Pm6 cells are in direct contact with the extracellular matrix, as evidenced by close associations with the pericellular layer. Initial separation of the larval grinder cuticle from the pm6 cell occurs by 5 minutes post-PC (**Fig 6B**), with the appearance of a thin, electron-lucent space between the basal surface of the L4 cuticle and apical surfaces of the pm6/pm7 cells (arrow in **Fig 6B**). At 10 minutes post-PC (**Fig 7A**), this space between pm6/pm7 and the L4 cuticle is wider and filled with an electron-dense material that fits to the basal side of the crests and troughs of the larval teeth. Also, the pericellular layer (layer 5) can no longer be identified, whereas layers 1–4 remain intact. We interpret the increased density of the region of separation between the L4 grinder and pm6/pm7 and the dissolution of the pericellular layer as the consequence of dense core vesicle exocytosis, which may result in release of lytic enzymes that facilitate extracellular structural breakdown. At 10 minutes post-PC, there is a pronounced accumulation of dense core vesicles in pm6/pm7 subjacent to the extracellular space, suggesting further secretion is underway (**Fig 7A**).

Dense core vesicles are the majority in EP I, followed by crescent-rimmed clear core vesicles and clear core vesicles. From top to bottom of the field of view, animal’s anterior to posterior axis. Scale bar = 1 μm.

At 15 minutes post-PC (**Fig 7B**), the L4 pharyngeal cuticle is intact from layers 1–3, but layer 4 has changed. It is transformed into diffuse, electron-dense blebs whose basal side is composed of cloudy and perforated matrix components. Presumably, this degradation matrix harbors fragments of the pericellular layer and layer 4.

**Fig 8:**
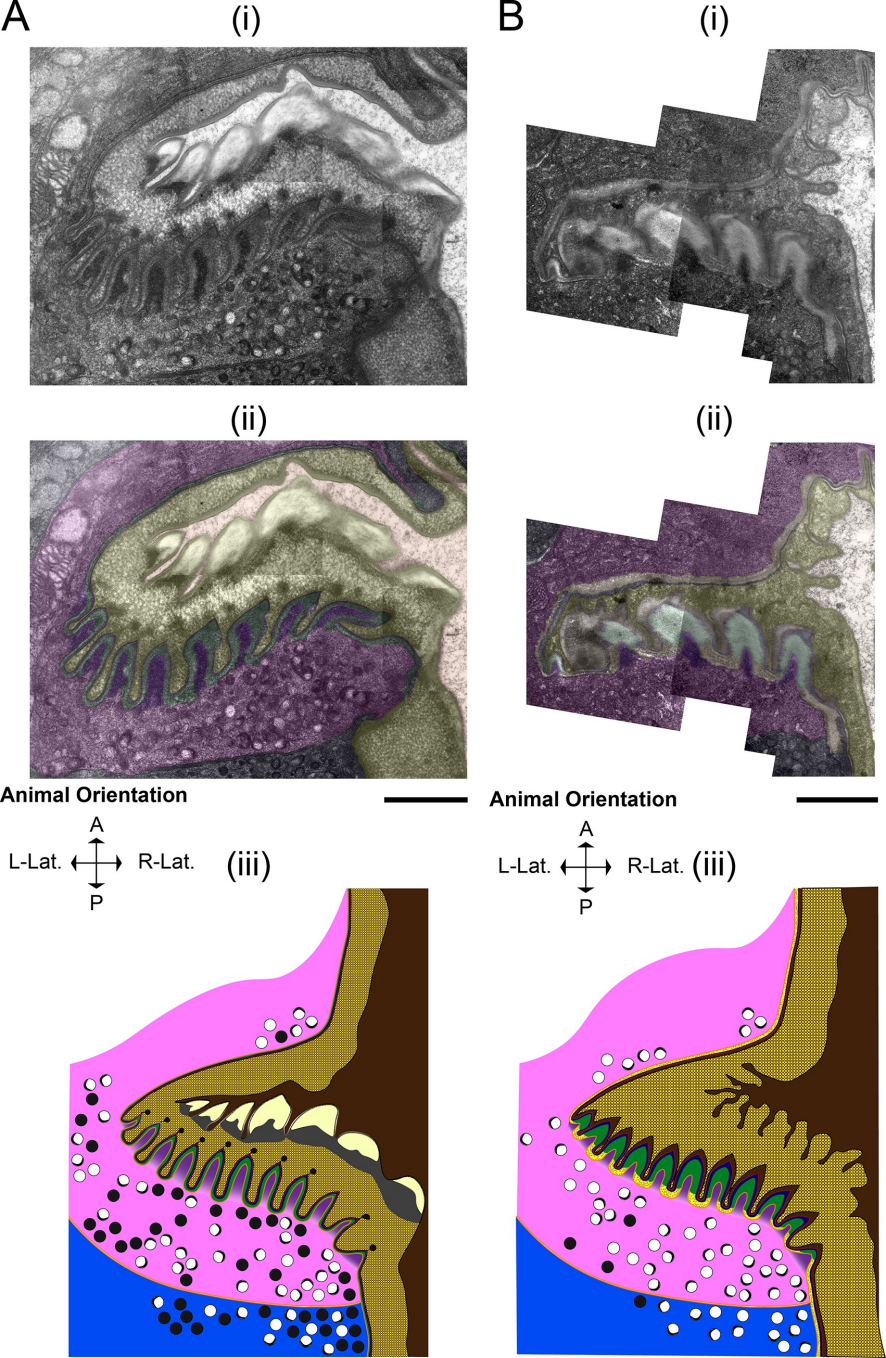
Layers 3–5 of the L4 grinder are fully dissolved as the adult grinder forms. (A) 30 minutes post-PC and (B) 60 minutes post-PC. (i) TEMs of the terminal bulb (ii) Pseudocoloring. Light brown denotes the terminal bulb lumen, yellow denotes the solubilizing L4 pharyngeal grinder, red denotes the lining the new adult luminal layer, blue denotes layer 2, green denotes layer 3, gold denotes layer 4, purple denotes pm6 papillae, pink denotes pm6, and navy denotes pm7. (iii) Cartoon representation using the color scheme used in ii, with the addition of plasma membranes denoted by orange. Anterior is up. Scale bar = 1 μm.

At 30 minutes post-PC (**Fig 8A**), layer 4 is further broken down, as evidenced by depletion of the electron-dense blebs, and the distance between the apical end of pm6/pm7 and the remnants of the L4 grinder is increased.

**Fig 9:**
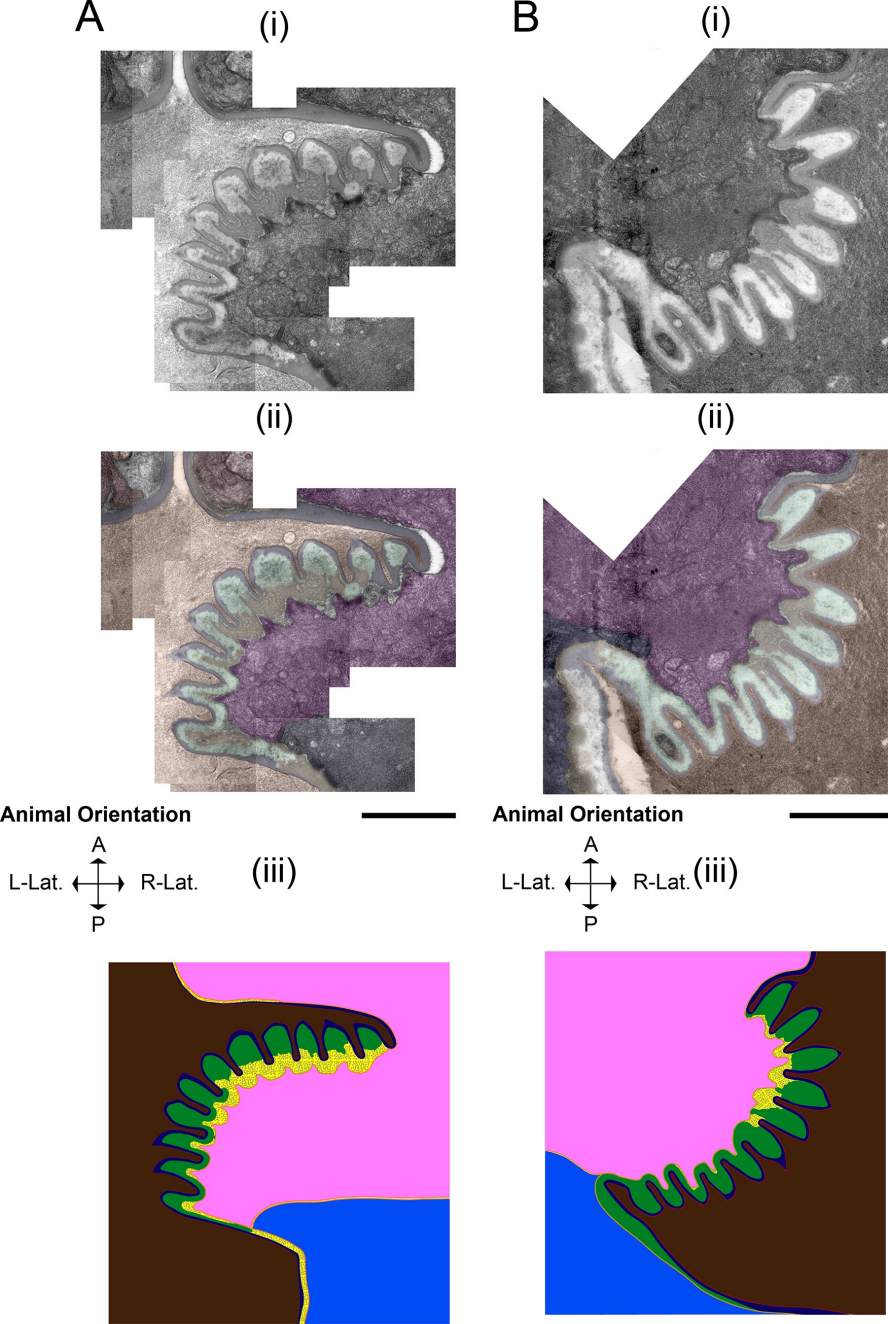
The formation and maturation of the adult grinder. (A) 2.5 hours post-PC (B) 1st day adult. (A and B) 2.5 hours post-PC and 1st day adult, respectively. (i) Transmission electron micrograph of the terminal bulb. (ii) Pseudocoloring. Light brown denotes the terminal bulb lumen, red denotes the lining the new adult luminal layer, blue denotes layer 2, green denotes layer 3, gold denotes layer 4, neon green denotes the pericellular layer, purple denotes pm6 papillae, pink denotes pm6, and navy denotes pm7. (iii) Cartoon representation using the same color scheme used in ii, with the addition of orange, which denotes plasma membranes. Vesicles are not shown, but sparse appearances of clear core are evident. Anterior is up. Scale bar = 1 μm.

At 60 minutes post-PC (**Fig 8B**), the L4 pharyngeal cuticle is nearly fully solubilized and appears amorphous, with only layers 1 and 2 still discernible. Video microscopy of a young adult animal exiting lethargus shows that the remnant of the L4 pharyngeal cuticle is swallowed into the intestine, coinciding with pharyngeal pumping-resumption (**S2 Video**). Our TEM data show no evidence of the L4 cuticle in the terminal bulb lumen of a young adult animal at 2.5 hours post-PC (**Fig 9A**). In summary, these results indicate that L4 cuticle digestion progresses from its basal side toward its luminal side but spares layers 1 and possibly layer 2, which are swallowed with the resumption of pharyngeal pumping. The persistence of layer 1 to enzymatic degradation during the molt suggests that it is made up of material that can resist lysis, such as functional amyloid and/or chitin or chitin metabolites. To better understand these mechanisms, it will be important in the future to determine which layers are composed of chitin and of which are composed of protein.

### The adult cuticle is built outside the cell by sequential addition of material to its basal side

Adult cuticle assembly occurs concurrently with larval cuticle degradation. At 10 minutes post-PC (**Fig 6C**), as dense core vesicles predominate in the cytoplasm of pm6-7 cells, we cannot yet discern a nascent adult grinder. However, by 15 minutes post-PC (**Fig 7B**), the nascent adult grinder is clearly recognizable and is made up of the three most apical layers, the luminal layer, layer 2, and layer 3. Interestingly, we observe semi-circle crowns of electron-dense material at the apical tip of each nascent adult grinder tooth. At 30 minutes post-PC (**Fig 8A**), these crowns take on a more rounded appearance and are separated from the tip of the adult teeth; they are enmeshed within the degrading layer 4 matrix of the L4 grinder. We speculate that these blobs, which disappear by 60 minutes post-PC (**Fig 8B**), mark the location at which each of the adult teeth is then built. At 30 minutes post-PC (**Fig 8A**), layer 3 is widened relative to 15 min post-PC (marked in green in **Fig 7B and Fig 8A**) while the luminal layer and layer 2 remain unchanged. Both at 15 min post-PC and at 30 min post-PC, the L4 matrix occupies the crevices between the newly formed adult grinder teeth.

At 60 min post-PC (**Fig 8A**), the adult grinder matrix contains four of five grinder layers, but the pericellular layer 5 is not yet apparent. Moreover, at 60 minutes post-PC, layers 1 through 4 appear to take on adult characteristics. Namely, layer 3 and layer 4 have a speckled granite and flecked marble patterning, respectively; layer 3 changes from high electron-density at 30 min post-PC to low electron-density at 60 min post PC, suggesting mineral (perhaps calcium) deposition has occurred (**Fig 8A and 8B**). Although, the pericellular layer is not seen at 60 minutes post-PC, pm6 electron-dense projections remain within the core of each adult grinder tooth (marked in purple in **Fig 8B**).

In a young adult animal at 2.5 hours post-PC (**Fig 9A**) and following pumping resumption, the adult grinder extracellular matrix is complete. The pericellular layer is present, indicating that it had formed sometime between 60 minutes post-PC and 2.5 hours post-PC. At 2.5 hours post-PC, we observe bacterial lysates within the lumen, indicating that the animal is awake and eating prior to fixation. The fully formed, young adult grinder at 2.5 hours post-PC is indistinguishable from the grinder of a day-1 adult (**Fig 9B**). At both time points, there are intact muscle striations in pm6/pm7 cells. At 2.5 hours post-PC and at the day-1 adult stages, several mitochondria are positioned below the grinder, but we observed only one mitochondrion during lethargus, at the five minutes post-PC stage (**Fig 9A and 9B**). This difference in mitochondrial density suggest differences in pharyngeal cell metabolic demand between the lethargus and adult stages.

A summary of our observations can be found in **Table 3**.

**Table 3:** Summary of observations of pm6, pm7, and the larval and adult grinders during the L4-to-adult developmental progression. Grey-shading indicates lethargus.

Stage	PM6/PM7	L4 Grinder	Adult Grinder
L4.7	Typical muscle cell properties; uninterrupted striations below L4 grinder; some dense core and clear vesicles.	Fully intact, five layers clearly visible: Luminal (1), 2, 3, 4 and 5 (pericellular.)	Absent
PC = 5	Sarcomere disruption at the interruption zone (IZ) beneath the plasma membrane facing grinder; Marked increase in DCVs; some clear core vesicles; One mitochondrion	Translucent space appears below layer 5 indicating grinder separation. Layers 1–5 intact.	Absent
PC = 10	Sarcomere disruption at IZ; Increase in DCVs; some clear vesicles. No mitochondria.	Layer 5 has dissolved and translucent space is filled with electron dense material. Layers 1–4 are intact.	Absent
PC = 15	Sarcomere disruption at IZ; DCVs persist; Marked increase in clear core vesicles and crescent-rimmed clear core vesicles. No mitochondria.	Layer 5 fully dissolved, layer 4 morphology has changed to diffuse, electron-dense blebs whose basal side is composed of cloudy and perforated matrix components (i.e. degradation matrix). Layers 1–3 intact.	Layers 1–3 appear. Semi-circle crowns of electron-dense material at the apical tip of each tooth.
PC = 30	Sarcomere disruption at IZ; Abundance of DCVs, clear core vesicles and crescent-rimmed clear core vesicles. No mitochondria.	Layer 4 further dissolved and space widens between pm6/pm7 and grinder. Layers 1–3 intact.	Layer 3 widens and becomes electron-dense; crowns become rounded, separate from the tip of the adult teeth and become enmeshed within the degrading layer 4 matrix of the L4 grinder.
PC = 60	Sarcomere disruption at IZ; Abundance of DCVs, clear core vesicles and crescent-rimmed clear core vesicles. No mitochondria.	Only layers 1 and 2 visible.	Layers 1–4 evident, pericellular layer not yet formed. Layer 3 displays a speckled granite appearance; layer 4 has a flecked marble patterning; layer 3 appears more electron-light, suggesting mineral deposition.
Adult PC = 150	Typical muscle cell properties; little to no vesicular activity and those present are clear vesicles; Several mitochondria positioned below grinder.	Larval grinder absent. S2 Video suggests that the layers 1 and 2 are swallowed into the intestine.	Fully intact, five layers clearly visible.

### Potential mechanisms of cuticle construction in the vicinity of cuticle degradation

Remarkably, the process of grinder formation occurs mere nanometers posterior to the synchronous process of the prior stage grinder dissolution. We offer several theories that are not mutually exclusive as to how this can occur. The first theory is that lytic enzymes are released into the luminal space after separation of the L4 cuticle from PM6/7 but before the formation of the new adult grinder luminal layer. The luminal layer of the new adult cuticle, which forms between 10 and 15 minutes post-PC, could be impermeable to the lytic enzymes released previously, protecting the nascent cuticle from degradation. This theory requires an explanation as to how the luminal layer is impervious to lytic enzymes but is consistent with our observations that the luminal layer of the L4 grinder is not digested, but rather is swallowed at the end of lethargus. Resistance to lytic digestion could occur if the luminal layer were composed of material such as functional amyloid that is inherently protease resistant [32], or hardy chitin or chitin metabolite. Alternatively, the luminal layer may contained inhibitors to lytic enzymes.

The L4 and adult cuticles appear to be scaled morphological replicas of one another, suggesting that the chemical composition of the grinder does not change with each iteration. However, it is possible that, at the molecular level, lytic enzymes released at each molt are unique and are dependent on the animal’s life stage. According to this theory, lytic enzymes released during L4 lethargus are active against L4 cuticle components, but are inactive against the adult cuticle components.

The focus of our study parallels that of Sedlak and Gilbert, who visualized cuticle shedding and formation in the tobacco hornworm, *Manduca sexta*. *Manduca* cuticle is made up of chitin, much like the cuticle lining the *C*. *elegans* pharynx [33]. At the beginning of the hornworm molt, plasma membrane plaques––electron-dense aggregations located at the tips of microvilli on the apical surface of epidermal cells––are attached to the endocuticle, the innermost layer of the larval hornworm covering. 2–3 days later, coated vesicles fuse at the microvilli bases, whereas at day 4, only clear core vesicles are observed. Separation of the endocuticle from the plaques occurs between days 6 and 7 in a process termed apolysis. Coincident with apolysis, electron-dense, filamentous aggregates––termed ecdysial droplets––appear in the space between the epidermal cells and the larval endocuticle [34]. Although the *M*. *sexta* molt occurs over a period that is close to 50 times longer than the *C*. *elegans* molt, this separation event in *Manduca* is reminiscent of the dissociation of the *C*. *elegans* grinder from the underlying pm6 myoepithelial cell at 5–10 minutes post-PC, as the accumulation of an electron-dense substrate between pm6 and the larval cuticle precedes cuticle digestion and new grinder formation. Taken together, these data suggest that at the cellular level, part of the ecdysozoan apolytic strategy is the coordinated exocytosis of lytic actors between the cell and the former cuticle.

### Astacin metalloprotease NAS-6 is required for digestion of the L4 grinder

The rapid degradation of the L4 grinder led us to hypothesize that secreted proteases were trafficked during the early stages of lethargus. The *C*. *elegans* genome codes for 40 secreted metalloproteases in the astacin class [35]. Remarkably, 23 of these astacin proteases show transcriptional oscillation during larval development [36] and 16 are up-regulated 30 minutes post-PC in L4 lethargus [16], suggesting that they play a role in the molt. A subset of astacin metalloprotease genes are expressed in pharyngeal muscle, suggesting that they function specifically in pharyngeal cuticle turnover.

The astacin metalloprotease NAS-6 shows cyclical mRNA expression during larval development and is expressed in the pharynx [35, 36]. Moreover, *nas-6* mutant animals grow at a stunted rate, and abnormal grinders are visible at the light microscope level [19]. We therefore used TEM to characterize these defects at the ultrastructural level.

**Fig 10:**
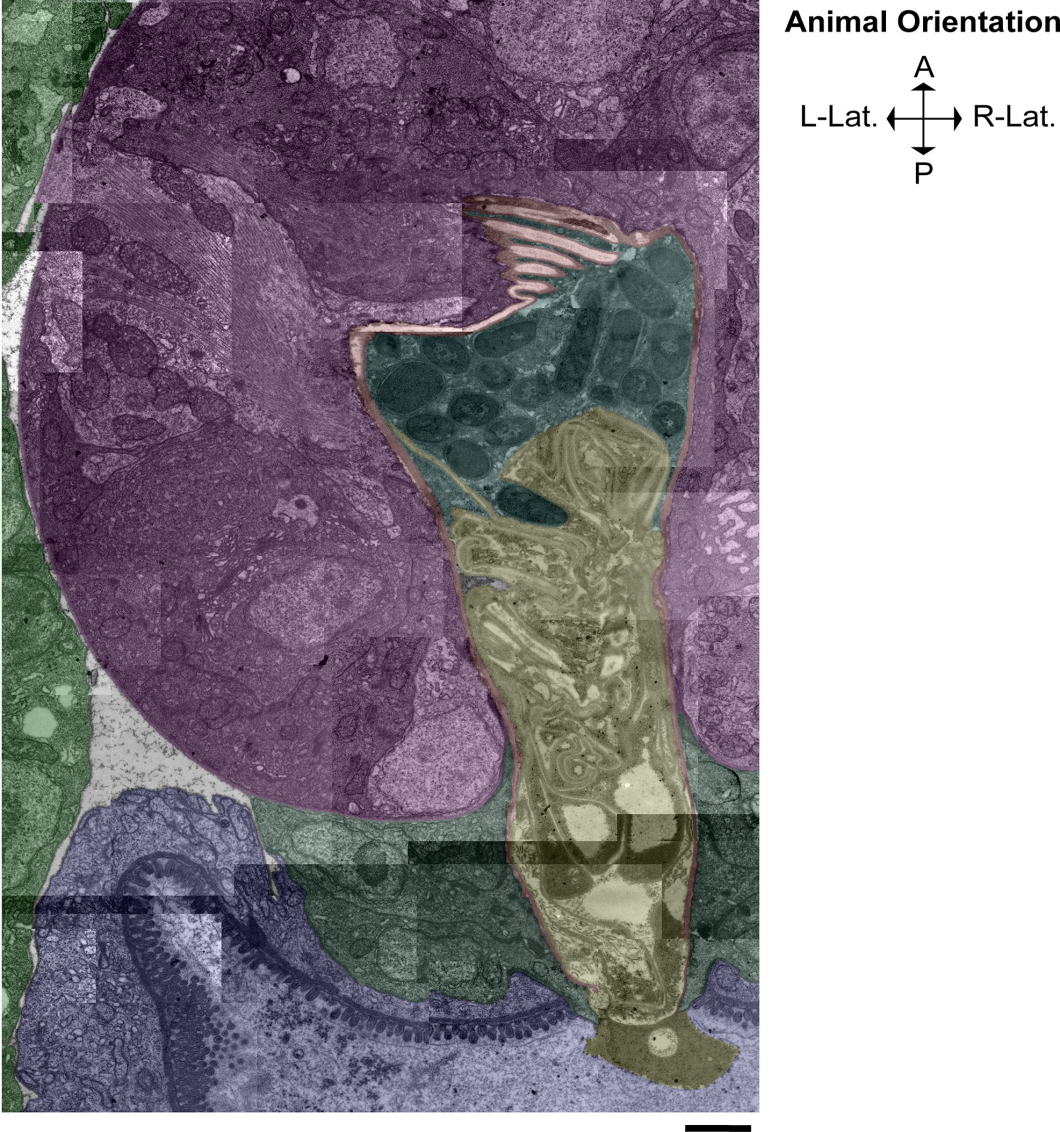
The astacin metalloprotease NAS-6 is required for digestion of the larval cuticle. Light green denotes hypodermal cells, pink denotes the terminal bulb, red denotes the adult grinder and pharyngeal cuticle lining, cyan denotes the terminal bulb lumen space where several *E*. *coli* bacteria are trapped, yellow denotes the ecdysed L4 cuticle, forest green denotes the pharyngeal-intestinal valve cells, and blue denotes the intestine. Regions not colored indicate the pseudocoelomic cavity. This micrograph of a *nas-6* mutant shows that the L4 cuticle was not digested and the alimentary passage was occluded by the undigested L4 cuticle, trapping intact bacteria in the terminal bulb and impairing mechanical disruption of bacteria. Anterior is up. Scale bar = 1 μm.

We observed the terminal bulb lumen of a *nas-6* mutant adult animal which contained intact *E*. *coli* bacteria as well as dissociated larval pharyngeal cuticle and components of the grinder. The detached L4 pharyngeal cuticle appeared intact but physically incapable of passing through the posterior end of the pharynx into the intestinal lumen. In contrast to the old L4 cuticle, which appeared largely undigested, the adult grinder appeared normal as intact adult grinder teeth and cuticle lined the entirety of the pharyngeal luminal space (**Fig 10**). Thus, we conclude that NAS-6 is required for the dissolution of the L4 grinder but not the formation of the adult teeth.

Since in *nas-6* mutants, the L4 grinder dissociates from pm6 muscle cells, it is likely that other lytic actors facilitate initial cleavage of the L4 cuticle, and that the vesicular aggregation in the separation space between 5–10 minutes coincides with NAS-6 release and action. Astacin metalloproteases, such as NAS-6, are produced as inactive proenzymes, necessitating cleavage of a prosequence to initiate enzymatic activity [37]. Thus, we propose that between 5–10 minutes post-PC, NAS-6 is among the lytic enzymes released from dense core vesicles, and that between 10–15 minutes post-PC, NAS-6 is activated by prosequence cleavage to begin cuticle disassembly.

### The aged grinder is worn down and ineffective

In the presence of food, adult pharynxes pump almost continuously at a rate of 250–300 pumps per minute [38]. Therefore, the pharyngeal grinder is subject to considerable mechanical stress throughout the life of the worm. In addition, the grinder functions to macerate bacteria, which produce lytic enzymes, resulting in exposure of the grinder to enzymes that degrade proteins and carbohydrates.

**Fig 11:**
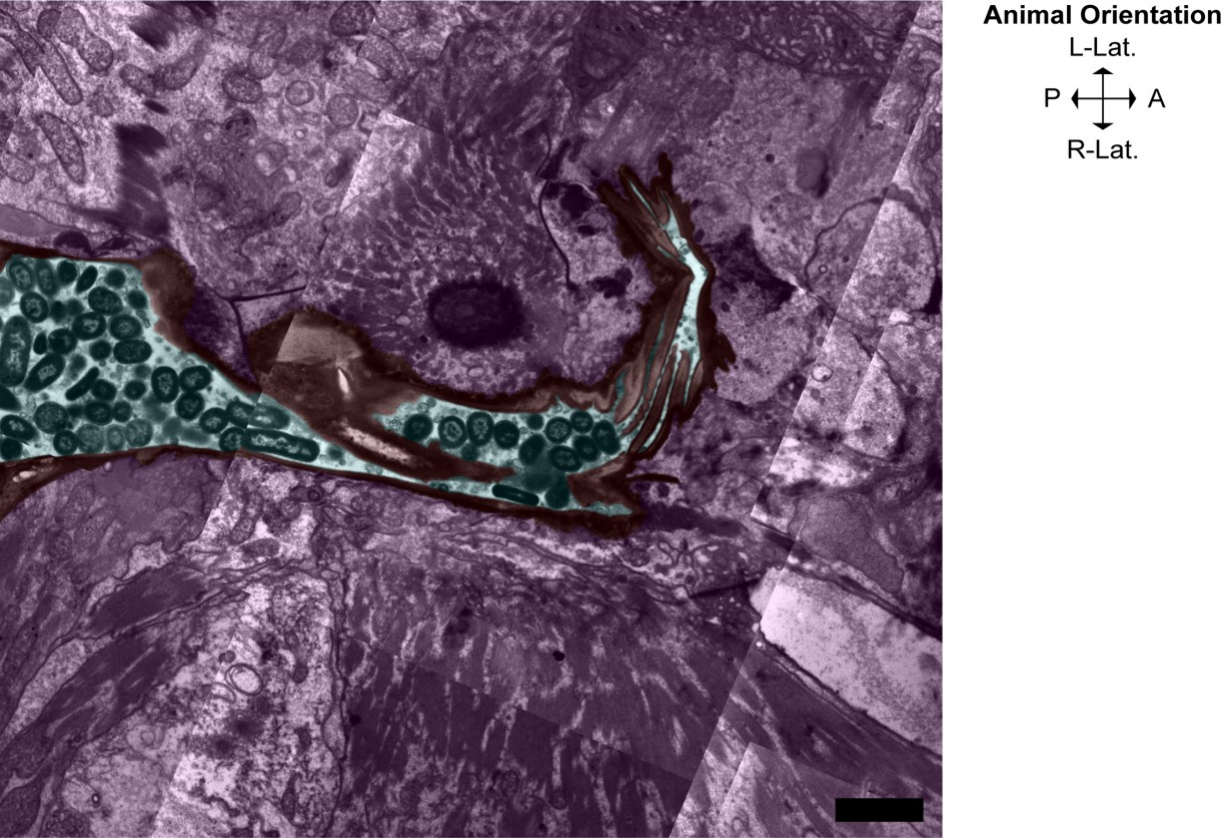
The aged grinder is worn down and permissive of transport of intact bacteria to the intestine. Pseudocoloring of a day-8 adult animal. Pink denotes the pharyngeal cells of the terminal bulb, red denotes the worn, adult grinder and pharyngeal cuticle, and cyan denotes the terminal bulb lumen and part of the intestinal space entrance (left,) where several intact *E*. *coli* bacteria are seen in close vicinity to the grinder teeth. Anterior is to the right. Scale bar = 2 μm.

The repetitive mechanical and chemical stress during feeding would presumably lead to deterioration of the matrix over time, much as human teeth wear down with age. We investigated whether or not aged animals maintain the integrity of the pharyngeal cuticle by visualizing the pharynx of a day-8 adult by TEM (**Fig 11**).

We learned two things by observing day 8 grinders. First, we observed that the teeth did not grow with age, even though adults continue to grow between day 1 and day 8 of adulthood. The day-8 adult grinder retains the same scale as a day-1 adult grinder, indicating that the grinder is only replaced during larval molts. Second, the day-8 adult grinder was significantly worn down. Adult grinder teeth were irregular, and contained notched edges, perhaps due to repetitive impacts of the other grinder teeth. The structural damage sustained by the aged grinder likely renders it dysfunctional, as evidenced by several intact bacteria seen within the pharyngeal terminal bulb lumen and by proliferation of intact bacteria in the proximal intestinal lumen. This age-dependent deterioration has been previously documented [39] and this dysfunction of the pharyngeal grinder may contribute to the eventual demise of the animals, perhaps via colonization by bacteria, as previously proposed [40].

## Supporting information

S1 Fig The *C*. *elegans* grinder is a specialization of the *C*. *elegans* pharyngeal cuticle, located in the medial part of the terminal bulb. (A i) A cartoon representative of a day-1 adult animal. (A ii) Magnified view of boxed region in (i). Pharynx muscle cells, denoted in crimson, synthesize and secrete the extracellular matrix components that form the pharyngeal cuticle, denoted in black, which lines the alimentary tract from the procorpus through the terminal bulb. The pharyngeal-intestinal valve (VPI) cells are shaded in green and are denoted with an arrow. (B) A 3D cartoon of a day 1 adult animal, with a blue rectangle depicting the approximate angle at which each specimen was cut longitudinally (the cutting plane). The animal is facing the viewer at a 45º angle and the pharynx is shown in the head/neck region in crimson. Scale bars = 10 μm.
(TIF)

S2 Fig Transmission electron micrograph montage of the medial terminal bulb region in a first day adult animal. Red line denotes representative whole grinder width measurement. Left insert: Orange and blue lines denote individual tooth width and height measurements, respectively. Right insert: All lines denote representative width measurements for individual grinder layers taken along the entirety of the pharyngeal cuticle in the terminal bulb, as follows: red, layer 1 (luminal layer,) blue, layer 2, green, layer 3, yellow, layer 4, and light green, layer 5 (pericellular layer). From top to bottom, the animal’s posterior to anterior axis. Montage scale bar = 1 μm, Insert scale bars = 200 nm.
(TIF)

S1 Video Time-lapse video (140 minutes) of L4 grinder dissolution and adult grinder formation. (MP4)

S2 Video Video of the L4 pharyngeal cuticle and grinder being swallowed into the proximal intestinal luminal, immediately after L4 lethargus and pumping resumption. (MOV)
