# Supplementary figures and images for: Teething during sleep: Ultrastructural analysis of pharyngeal muscle and cuticular grinder during the molt in Caenorhabditis elegans
Source: PLoS One. 2020 May 20;15(5):e0233059. doi: 10.1371/journal.pone.0233059 (PMC7239488; doi:10.1371/journal.pone.0233059)

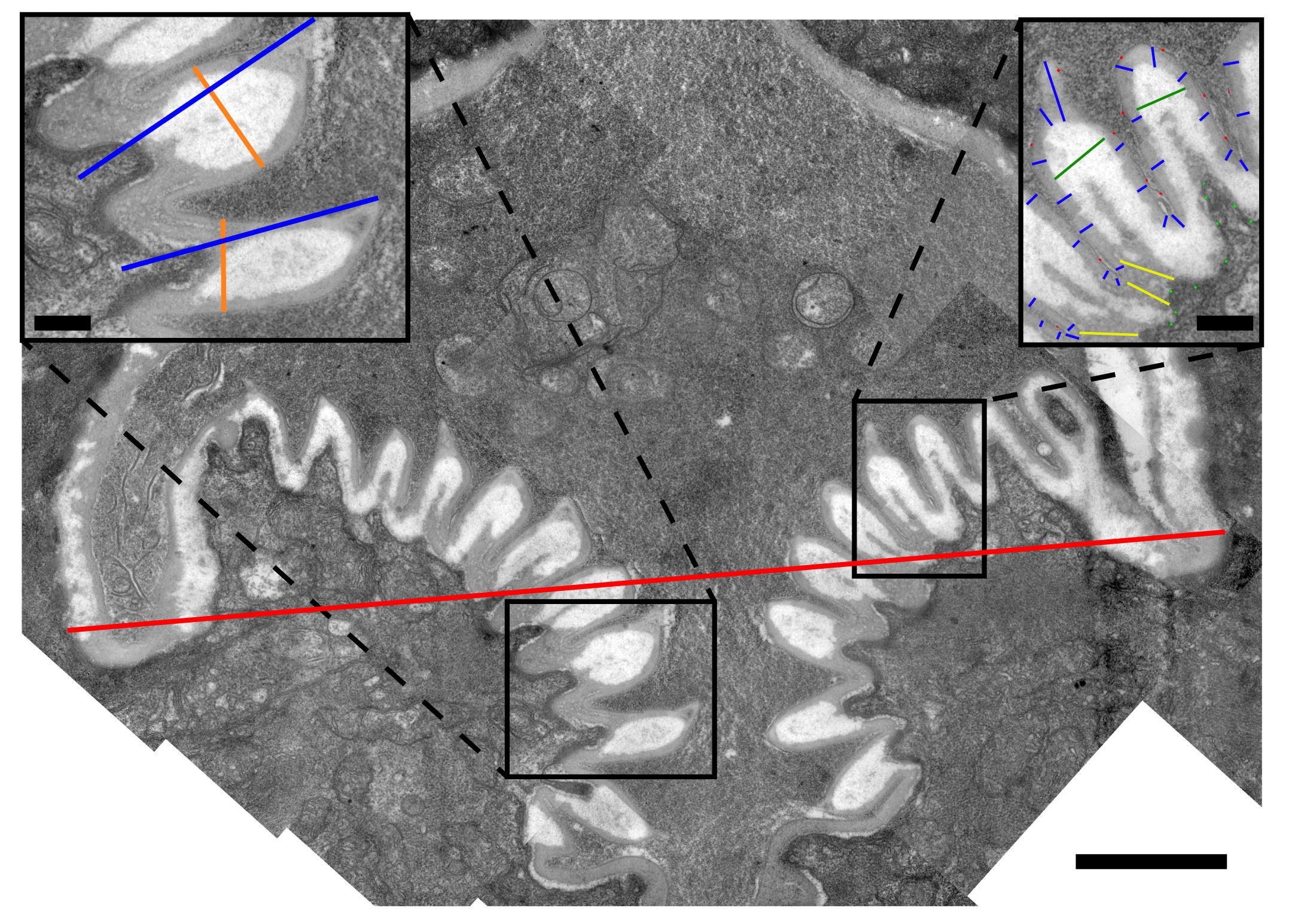

Supplement: S2 Fig — Red line denotes representative whole grinder width measurement. Left insert: Orange and blue lines denote individual tooth width and height measurements, respectively. Right insert: All lines denote representative width measurements for individual grinder layers taken along the entirety of the pharyngeal cuticle in the terminal bulb, as follows: red, layer 1 (luminal layer,) blue, layer 2, green, layer 3, yellow, layer 4, and light green, layer 5 (pericellular layer). From top to bottom, the animal’s posterior to anterior axis. Montage scale bar = 1 μm, Insert scale bars = 200 nm. (TIF) [file pone.0233059.s002.tif]
